# Regenerative memory in time-delayed neuromorphic photonic resonators

**DOI:** 10.1038/srep19510

**Published:** 2016-01-19

**Authors:** B. Romeira, R. Avó, José M. L. Figueiredo, S. Barland, J. Javaloyes

**Affiliations:** 1 Centro de Electrónica, Optoelectrónica e Telecomunicações (CEOT), Departmento de Física, Universidade do Algarve, Campus de Gambelas, 8005-139, Faro, Portugal; 2 Institut Non-Linéaire de Nice, Université de Nice Sophia Antipolis, CNRS UMR 7335, 06560 Valbonne, France; 3 Departament de Física, Universitat de les Illes Baleares, C/Valldemossa km 7.5, 07122 Mallorca, Spain

## Abstract

We investigate a photonic regenerative memory based upon a neuromorphic oscillator with a delayed self-feedback (autaptic) connection. We disclose the existence of a unique temporal response characteristic of localized structures enabling an ideal support for bits in an optical buffer memory for storage and reshaping of data information. We link our experimental implementation, based upon a nanoscale nonlinear resonant tunneling diode driving a laser, to the paradigm of neuronal activity, the FitzHugh-Nagumo model with delayed feedback. This proof-of-concept photonic regenerative memory might constitute a building block for a new class of neuron-inspired photonic memories that can handle high bit-rate optical signals.

Self-feedback connections in neurons are common in the nervous system and are named autapses^1^,^2^,^3^,^4^. These synapses between a neuron and a branch of its own axon are ubiquitous and have been found in the neocortex and the hippocampus, to cite but a few^1^. Yet their purpose has remained uncertain. Recent reports suggest that autaptic transmission neurons are involved in the long-lasting response to brief stimulations^3^, and that this persistent activity has important implications in local feedback regulation^4^, and working memory. On the other hand, recent progresses in multidisciplinary fields including semiconductor physics, photonics, computing and networking yielded the possibility to emulate some elementary functions of the brain using neuromorphic systems^5^,^6^,^7^,^8^. The central goal is to reproduce neuronal synapses by interconnecting thousands of neuron-like elements^9^. While a lot of attention has been dedicated to the network architecture of neuromorphic systems^7^,^8^, almost no attention has been paid to the self-feedback autaptic connections providing localized persistence of neuronal activity.

In this work, we implement a regenerative memory in a bio-inspired time-delayed neuromorphic photonic resonator that exhibits long-lasting responses and short transients to brief stimulations. The information is stored in the temporal configuration as an ensemble of coherent structures which possess all the functional properties of localized states (LS) often observed in spatially extended and out-of-equilibrium nonlinear system. These LS can be explored in regenerative memory and information storage. Because we use light at telecommunications wavelengths (~1.55 um) for the regeneration of the spike-neuron signals, we anticipate the development of fast (Gb/s) neuron-inspired optical storage interconnects and signal processing applications. We reduce our proof-of-concept experimental system to the prototypical FitzHugh-Nagumo (FHN) model, a paradigm of neuronal response, complemented with a self-feedback autaptic connection. This analysis bridges our physical photonic resonator with the biological neuron and extend our findings to other biological, physical and engineering systems.

A lot of interest was devoted during the past decades to the effects of communication time delays^10^,^11^ and how they can influence the synchronization dynamics between distant coupled neurons. For instance, it was shown that dynamical systems mimicking coupled neurons^12^,^13^,^14^,^15^ exhibit, instead of a steady state, stable periodic pulsating regimes where the two neurons may release energy in anti-phase, the period being related to the delay value. Following the seminal work of Ikeda^16^, the concept of pattern memorization in time delayed bistable systems was addressed in opto-electronic systems, see for instance^17^,^18^.

Departing from these works, our experiment involves an excitable element as the nonlinear node. Thanks to this crucial difference, the present approach benefits from the robustness and self-healing properties which are typical of neural signals and do not exist in bistable systems. Specifically, the excitable response of the nonlinear node guarantees a strong and well-defined all-or-nothing pulse response almost identical in shape and duration to any supra-threshold incoming signal, which results in a built-in reshaping and healing functionality for incoming data stream.

The presence of time delays in dynamical systems is well known to induce temporal oscillations. However, it may also have a much deeper influence as even a *single* delayed equation is akin to an infinite dimensional dynamical system. In particular, important conceptual links between partial differential equations (PDE)s and delayed differential equations (DDE)s exist^19^. As such, one is lead to wonder if the rich dynamics found in spatially extended systems, and in particular their ability to store information^20^ in the form of Localized Structures (LS), could also exists in the temporal output of a *single* neuron with a delayed coupling representing an autaptic connection as seen in (Fig. 1a).

Localized states have been widely observed in nature in systems like granular media^21^, gas discharges^22^, semiconductor devices^23^, reaction-diffusion systems^24^, fluids^25^, convective systems^26^ and optical cavities^27^,^28^,^29^,^30^. Localized structures appear in a dissipative environment as attractors, i.e. stable solutions towards which the system will evolve spontaneously from a wide set of initial conditions^31^,^32^ making them intrinsically robust and allowing for complex interactions. While in principle fundamentally related to the existence of spatial degrees of freedom, LS analogues have recently been observed in periodically modulated and autonomous delayed dynamical systems^30^,^33^.

The self regenerative orbits presented in this work in the context of delayed systems verify all the conditions necessary to support their interpretations as temporal LS. Yet in delayed systems, most temporal traces are akin to periodic solutions with a period close the value imposed by the time delay *τ*. In particular, we stress that for given number of LS that could coexist within a time delay, any translation of one or several LS would give rise to another limit cycle. As LS are indeed independent, one expects to find for each LS a translation mode corresponding to a neutral mode of the underlying limit cycle. In this respect if LS are to be found in delayed systems, they shall correspond to highly non-generic regimes in which one may find an infinity of stable coexisting limit cycles, each one possessing a number of neutral translation modes equal to the number of LS in the solution.

## Results

### The neuromorphic photonic resonator: experiment and model

**Figure 1:**
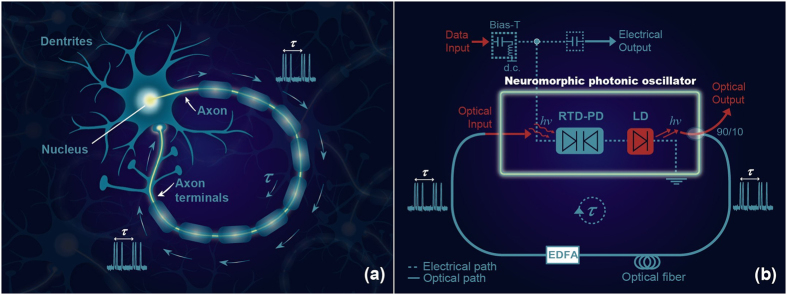
Neuron-inspired regenerative memory with autapic connection. (**a**) Diagram of a neuron with a self-feedback effect due to the presence of an autapse. (**b**) Schematic of the equivalent time-delayed neuromorphic photonic resonator in which the optical output is re-injected after a time delay due to the propagation into an optical fiber. (RTD-PD: resonant tunneling diode photodetector; LD: laser diode; EDFA: erbium doped fiber amplifier)

Our time-delayed system consists of a nanoscale negative differential conductance device based on a resonant tunneling diode photodetector (RTD-PD) set in an excitable regime and driving an on-chip laser diode (LD). Although electronic neuron-like semiconductor microstructures^34^ have also been proposed, they operate at rather low-speeds (of the order of 20 kHz) and do not possess optical input/output ports as the neuromorphic system proposed here. Neuromorphic responses were achieved in lasers^35^, nanolasers^36^ and alternative opto-electronic configurations^37^,^38^ and could give rise to alternative implementations. The schematic diagram of our experimental neuromorphic photonic memory is depicted in (Fig. 1b). The RTD response drives the laser diode such that a pulse in the RTD current produces an optical pulse. The RTD-PD provides a non-monotonic Current-Voltage (I-V) curve with a region of negative differential resistance, in which it behaves as an excitable system, as demonstrated in^39^.

**Figure 2:**
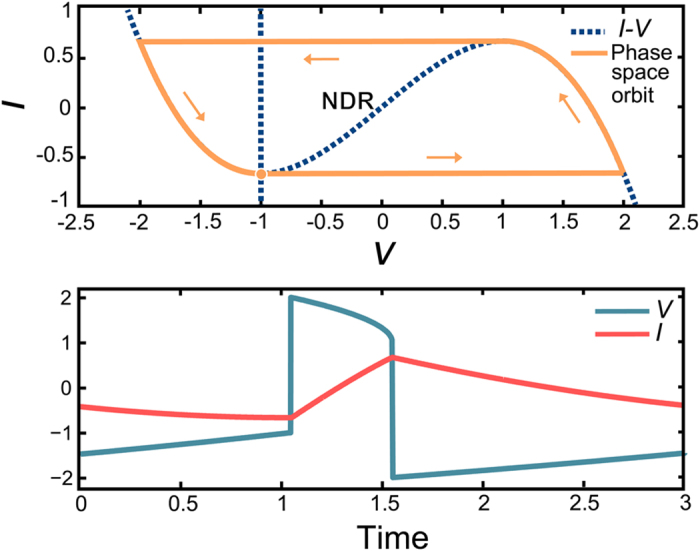
Excitable response of the FHN model. Top: Nullclines and phase space trajectory of the FHN model for *β* = 1. Bottom: Time domain traces of voltage (V) and current (I) for a single phase space trajectory

Excitability is a concept originally coined to describe the capacity of living organisms e.g. nerves^40^,^41^,^42^ or neurons to respond strongly to a weak external stimulus that overcomes a well defined threshold. If the system is perturbed from its rest state, it may relax back toward its steady state in two different ways. If the perturbation remains below a certain threshold, the relaxation is exponential. Above the threshold, the system has to perform a large orbit that involves the whole phase space topology before relaxing again towards the unique fixed point (the rest state). Such a relaxation is visible for instance in the lower panel of Fig. 2. These two widely different transient regimes toward a unique attractor are what defines the so-called excitability phenomenon. During its large excursion in phase space, the system cannot respond to another perturbation which defines incidentally the so-called lethargic time *T*_*l*_ as the temporal extent of the orbit. Well known in physiology, this refractory period corresponds physically to the fact that a large amount of energy is released during the excitable response and may be understood as the time the system needs to recharge before being able to release another response. In neurons, this period of time occurs during the re-polarization and the hyperpolarization of the membrane potential.

In order to operate our neuromorphic photonic system as a regenerative memory, an optical delay line provides the temporal buffer memory that will store the bits of information as light intensity pulses. The excitable response of the RTD ensures the regeneration and the rectification of the signal as a special nonlinear node along the propagation loop. An erbium doped fiber amplifier (EDFA) is employed in the loop to control the amount of feedback coupling and compensate for the losses incurred by coupling and decoupling light from the RTD-PD and LD dies (refer to Methods section). The light pulse re-injection into the RTD-PD triggers, after the round-trip in the fiber, a new electrical response in the nano-optoelectronic system thereby repeating the cycle with a period close to the optical pulse propagation time, *τ*, in the fiber.

A precise modeling of the experimental situation can be achieved within the framework of a dynamical model employing a Liénard equation^43^ describing the RTD-PD oscillator^44^,^45^ coupled to the single mode rate equations modeling the laser intensity and its population inversion (Methods section). Such an approach was shown to yield a quantitative agreement with the experimental results^39^. However, by assuming that the excitable response is slower than the relaxation oscillation frequency of the laser, one can adiabatically eliminate the laser intensity (*s*) that becomes slaved to the current (*i*) of the RTD. By expanding the nonlinear characteristic of the RTD at the center of the negative differential resistance, denoting *V* the deviation of the voltage and assuming it is antisymmetric, one may reduce exactly the underlying physical model to the FHN model with delayed feedback, making a complete link with our time-delayed neuromorphic photonic system and the paradigm of excitability (refer to Methods section).

The FHN model^41^,^42^ represents a simplified version of the theory developed by Hodgkin and Huxley^40^,^46^ to study how action potentials in neurons are initiated and propagated. The time delayed FHN model reads


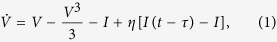



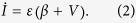


The stiffness parameter *ε* denotes the ratio of the time scale governing the slow (*I*) and the fast (*V*) variables while *β* is the bias parameter. We choose *β* > 0 without loss of generality. The influence of the delayed re-injection of light, proportional to the current in the RTD, is taken into account by the delayed term in Eq. (1). The amplitude of the delayed feedback is denoted *η*. For the sake of convenience we use the so-called form of non invasive feedback^47^. As such, the steady states of the FHN model are unchanged by the presence of feedback. In correspondence with the experimental situation, we re-inject the slow variable after a time delay *τ*, into the dynamics of the fast one as *I*(*t* − *τ*), yet very similar results were obtained in other situations, e.g. re-injecting *V*(*t* − *τ*) instead. If not otherwise stated the parameters are *ε* = 0.05, *η* = 0.18 and *τ* = 500. We included white Gaussian noise of variable amplitude *ξ* to model the stochastic processes occurring in our experimental neuromorphic photonic oscillator (refer to Methods section).

### Memory Operation

We start the analysis of the neuromorphic photonic oscillator by briefly recalling the main properties of Eqs (1) and (2) in the absence of delayed feedback. For values of the bias 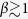, the unique steady state (*V*_*s*_, *I*_*s*_) = (−*β*, *β*^3^/3 − *β*) is stable. Yet close to *β* = 1, the system is excitable. For a sufficiently large perturbation the system emits an excitable orbit before going back to its rest point as depicted in Fig. 2. The duration of this orbit represents the refractory time *T*_*l*_. The largest the distance of the bias from *β* = 1, the higher the excitable threshold, that is, the minimal energy of the perturbation giving rise to the excitable response.

**Figure 3:**
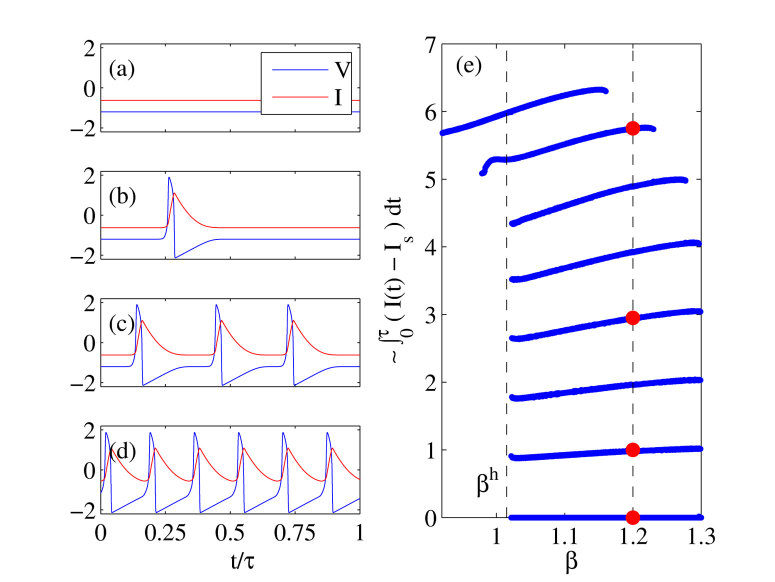
Numerical simulations of the memory operation as a function of the bias parameter. The blue curve represents V and the red curve represents I. Temporal time traces over a single period for various states of the regenerative memory (**a–d**) and multi-stability diagram of the coexisting solutions (**e**).In (**e**) we represent some norm of the solutions as the integral over one period of the deviation of the slow variable (*I* − *I*_*s*_) yielding upward pulses with a zero background. This integral is normalized to a value of 1 when there is a single pulse at *β* = 1.3. All the localized solutions becomes unstable close to *β*^*h*^ ~ 1.018 where the background get destabilized through an Andronov-Hopf bifurcation.

In contrast with previous works^13^,^15^, we are interested in the regimes in which the values of the time delay are large as compared to the lethargic time *T*_*l*_ which in our case is *T*_*l*_ ~ 3*ε*^−1^, see^48^ for more details. We demonstrate in Fig. 3 that Eqs (1) and (2) support in this case the storage of information. The presence of a weak delayed perturbation in Eqs (1) and (2) induces a multi-stability between an infinity of different temporal patterns that repeat themselves identically with a period close to the time delay *τ*. For a broad range of values of the feedback parameter *η*, for which the re-injection of the delayed excitable orbit after a time delay *τ* is sufficient to overcome the excitability threshold, a new, perfectly formed, excitable orbit gets regenerated. As such, the excitable orbit of the solitary FHN system, whose temporal extend is *T*_*l*_, becomes a binary unit of information *embedded* in a much longer periodic orbit of period *τ*. Such bits of information get perfectly regenerated after each period and since the mere condition for *perfect* regeneration is to overcome the excitability threshold, one foresees the signal healing properties and the robustness of this mechanism.

We exemplify in (Fig. 3a–d) various occurrences of such periodic regimes whose periods are close to *τ* and that are composed of 0, 1, 3 and 6 bits of information as embedded excitable responses within the time delay *τ*. We stress in (Fig. 3e) that all these regimes coexist between themselves for a wide range of the bias parameter *β*. Because these isolated temporal patterns are bistable with the uniform state and are also independent of the boundary conditions, i.e. the time delay value, and are attractors of the dynamics, they can be considered as the equivalent of Localized Structures in time delayed systems.

The inclusion of time delays in a dynamical system usually excite temporal oscillations in the form of Andronov-Hopf bifurcations found e.g. as a function of the strength of the feedback level of the duration of the time delay. Yet we stress that the results found in Fig. 3 can not be reduced to such mere delay-induced pulsations. The proof of that being that the homogeneous solution with *N* = 0 LS remains stable and still coexists with all the other aforementioned limit cycles.

We depict in Fig. 3e the norm of the various solutions as well as the maximal number of bits of information that can be stored for a given value of *τ*. Interestingly, each branch of solution corresponds to a well defined number of temporal LS. However, what is hidden in such a projection is that for a given number of bits, i.e. a given branch, an infinity of different arrangements and relative distances exists. The storage capacity of our regenerative memory can be understood intuitively. Since the temporal extension of the excitable orbit is defined by its lethargic time *T*_*l*_, the maximal amount of elements that can be stored in the time delay is the integer closest to *N* ~ *τ*/*T*_*l*_ = 8, in good agreement with numerics where we found *N* = 7 leading to 2^7^ = 128 (regular) configurations.

**Figure 4:**
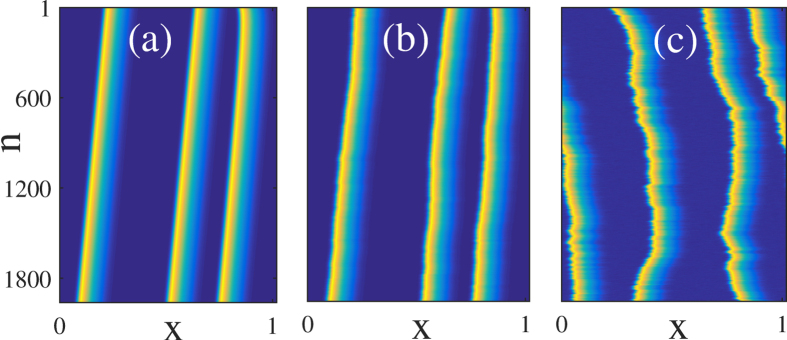
Numerically simulated space-time plots showing the evolution of the solution depicted in Fig. 3(c) with three LS for an increasing level of noise (the slow variable represented is I). In panel (**a**) *ξ* = 10^−3^ and the noise induced drift motion is barely visible over more than *θ* = 2000 periods. In panel (**b**) the uncorrelated random walk is more visible since *ξ* = 2 × 10^−3^ while in (**c**) the huge level of noise *ξ* = 5 × 10^−3^ is capable of inducing a complete loss of the signal integrity over a time scale of a few tens of round-trips.

A further proof of the mutual independence of the temporal LS can be found in a two dimensional pseudo-spatial representation depicted in Fig. 4 in which the relative motion of the various bits of information over longer time intervals are best observed. In this co-moving reference frame, the horizontal axis is a space-like coordinate that allows to localize the position of the pulses within a given round-trip while the vertical coordinate corresponds to the slow temporal evolution of the system over many round-trips. The mutual independence of these pulses, as demonstrated for instance by their uncorrelated random motion in the presence of noise in (Fig. 4c) confirms their nature of LS. Finally, the existence of the lethargic time induces an effective repulsion between nearest bits of data when they get too close, thereby ensuring signal integrity.

With respect to the storage of information in a neuromorphic excitable system with delayed retro-action, the present work heavily differs from^30^ first by its optoelectronic nature which has many advantages with respect to monolithic integration, possible coupling and multi-component inputs. In addition, our neuromorphic system is of the “resonator” type, whereas that of^30^ is of “integrator” type, see^49^ for more details. This peculiarity gives to our system a different set of computational properties. For instance, resonator neurons have a much shorter refractory time, which in the present context may result in much higher bit density in the memory. Furthermore, resonator neurons have the capability of selectively respond to periodic sub-threshold perturbation and therefore to act as tunable time-lag coincidence detectors, which integrator neurons cannot do.

**Figure 5:**
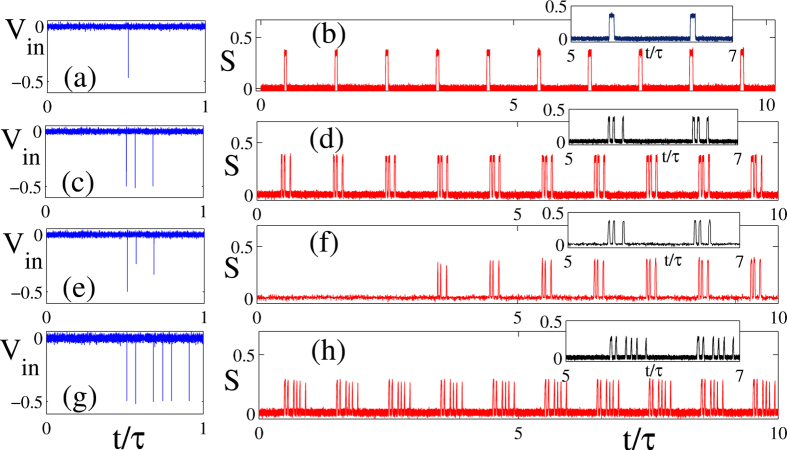
Experimental time traces of showing writing and storage of binary-coded data streams in a *τ* = 64 *μ*s cavity round-trip time. (Left) data streams input, and (right) laser photo-detected optical output. Sequence of (**a**,**b**) 1-bit, (**c**,**d**) 4-bit (1101), (**e**,**f**) the same 4-bit but with a degraded input which yields the same state in the memory. In panel (**f**) the absence of transient is visible and (**g**,**h**) a one byte (11011101) sequence.

We assessed experimentally the robustness of the writing and storage process by using the experimental setup shown schematically in Fig. 1b and by employing several temporal bit patterns, see Methods section. Figure 5(a,b) shows an example of complete regeneration using a single trigger event composed of one bit (1), where a single trigger is able to generate a perfectly formed train of identical pulses. The regeneration of a four-bit (1101) pattern in depicted in Fig. 5(c,d). As mentioned previously, the bits must be separated at least by the lethargic time *T*_*l*_ of the excitable system. In our case we found *T*_*l*_ = 0.5*μ*s so that with *τ* = 46*μ*s the “fiber storage capacity” is *τ*/*T*_*l*_ = 92, quite larger than in the numerics where the delay was kept small for numerical convenience. Moreover, it is worth noticing that triggering an identical bit sequence can be done with a strongly degraded initial pattern, as visible in Fig. 5(e,f). This demonstrates that our system performs single-pass-healing by restoring and self-adjusting the received bits to a fixed amplitude, confirming that the nature of the excitable response renders the regenerative memory almost insensitive (in a certain range) to the exact shape or the amplitude of the addressing pulses. One notices the almost complete absence of transient in Fig. 5(f). This is indeed a property of the utmost importance in our system. The localized bits of information can be perfectly written and erased in a single round-trip, at variance with the results of^29^,^50^ where transients representing tens of round-trip are necessary before a stabilization of the waveform. From an application point of view, our time-delayed neuromorphic photonic memory presents the extraordinary advantage of allowing the writing and the erasing of information at a rate comparable to the nominal reading rate. As such, we believe that the possibility to harness the unique properties of the excitable response to be of great importance and to cross boundaries between specific fields. We emphasize that the writing and storage process is extremely robust as shown in Fig. 5(g,h) where we show the stable regeneration of a complex pattern of 8-bits (11011101) over a time scale in the ms range.

**Figure 6:**
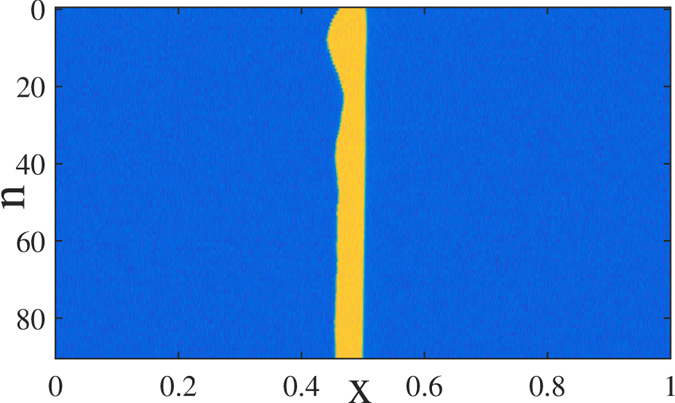
Experimental space-time plot showing the evolution of the solution depicted in Fig. 5(a,b) with a single LS. A transient on the rising front of the LS is visible over a time scale that corresponds to 50 round-trips, i.e. ~2 ms.

We present in Fig. 6 a space-time plot corresponding to the evolution of the solution depicted in Fig. 5(a,b). While the excitable orbit is almost perfectly regenerated after a single round-trip, one notices a remaining transient that affects the rising front of the LS. As this effect was not encountered in the FHN model, we infer that it is the result of the slow gain dynamics incurred by the presence of the EDFA in the experiment. Typical gain transient range from *τ*_*g*_ = 100 *μ*s to *τ*_*g*_ = 10 ms which is an excellent agreement with the transient found. Although the details and the shape of the re-injection pulse are to some extent irrelevant in order to trigger the excitable regeneration, its total energy is an important parameter. Because the precise amount of energy re-injected into the system evolves in time due to the slow gain of the EDFA, this induces some jitter on the timing of the rising (left) front of the pulse. Although very interesting from the point of view of the nonlinear dynamics, the additional features induced by a slow gain time scale could be seen as a limiting factor for the applicative potential of our RTD based optical memory. Yet, the need for signal amplification merely stems from the low coupling into the RTD photodetector window that can be widely optimized, rendering the presence of the EDFA unnecessary.

Experimentally, we achieved writing, storage and healing of binary-coded data streams. In order to fully test the robustness of our optical buffer memory and extend its capabilities, namely reproducible erasing of data and operation at high bit-rate signals, further work is currently underway in the design and fabrication of neuromorphic oscillators operating at higher frequencies and with optimized detection efficiencies. This can potentially improve the setup stability for a robust buffer operation by avoiding the EDFA and requiring much shorter optical fiber feedback loops.

We note that although the experimental conditions are identical for all the panels of Fig. 5, the individual pulse shape changes as a function of the number of LS in the round-trip. This is again an effect that we attribute to the EDFA slow amplification. In the steady regime, where *N*-LS are properly established in the round-trip, the EDFA works in the so-called saturated regime. Because the EDFA time scale is slower than the round-trip *τ*_*g*_ ≪ *τ*, the gain can be assumed constant within a time delay, an approximation usually performed successfully in Fiber mode-locked lasers. Importantly, this average saturated gain is a function of the number of LS in the round-trip. A larger number of LS implies a lower saturated gain and a lower amplification for each LS. This explains why the shape of the LS are different depending if we have a single or multiple LS. Actually, to compare the *N* = 3 LS solution with the *N* = 1 LS one should lower the EDFA gain in the latter case to take into account the lower amplification in the former one.

Finally, it is interesting to contrast our results with the one discussed in the case of the time delayed nonlinear opto-electronic oscillators based upon a Mach-Zehnder interferometer^37^, in particular Fig. 17 in^37^. Here a regular periodic train of irregularly separated pulses and bursts repeat, yet with a period close to *twice* the value of the time delay. As mentioned by these authors, the mechanism and the theoretical explanation behind these periodic self-pulsating was not easy to identify. We believe that the results in our manuscript and our interpretation of the dynamics as regenerative LS could maybe provide a framework for explaining the results of Peil *et al.*^37^, although the physical system presented is different.

### Bifurcation analysis

**Figure 7:**
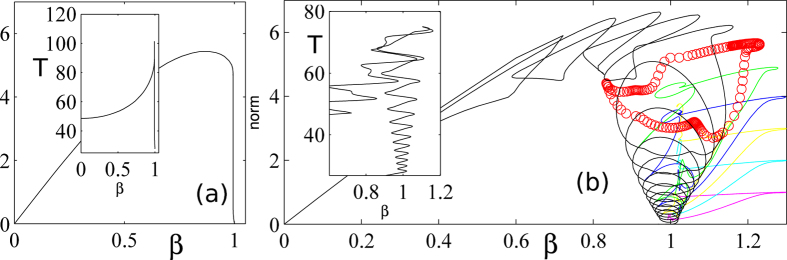
Bifurcation theoretical analysis of formation of multiple states of memory. (**a**) Amplitude of the periodic solutions and variation of the period along the branches of (inset) for the FHN system without feedback i.e. *η* = 0. (**b**) Same diagram with *η* = 0.18 the colors correspond to the branches of solutions with 1, 2, … 7, equi-spaced localized structures.

In this section, we shed some light onto the mechanism of formation of the multiple states of the memory by performing a bifurcation analysis of Eqs (1) and (2). It is instructive to recall briefly the main properties of the FHN model represented by Eqs (1) and (2), in the absence of delayed feedback. An Andronov-Hopf bifurcation occurs at β^*^ = 1 where small amplitude harmonic oscillations develop around the steady solution with an oscillation frequency 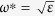, see the left inset in (Fig. 7a). These oscillations around the background solution rapidly develop into a large amplitude nonlinear limit cycle as soon as *β* deviates slightly from β^*^. This rapid blowing-up of the oscillation amplitude is famously known as a canard phenomenon^51^ and corresponds to a bifurcation being almost vertical, see (Fig. 7a). Importantly, in addition to the blow-up of the amplitude of the limit cycle, the period of the oscillations strongly deviates from the harmonic value found at the Andronov-Hopf bifurcation, see inset in (Fig. 7a). In this canard regime, the Eqs (1) and (2) represents a so-called relaxation oscillator^52^,^53^ whose period is proportional to the inverse of the stiffness parameter, i.e. in our case *T* ~ 3*ε*^−1^, see^48^ for more details. We stress that in this regime, the only stable solution of Eqs (1) and (2) consists of this nonlinear limit cycle. However, in the excitable regime, found for values of *β* close but above β^*^, the lethargic time *T*_*l*_ and the shape of the excitable response identifies with the period and the waveform of the relaxation oscillator in the oscillatory regime.

As depicted in Figs 3 and 4, the existence of complex spatio-temporal patterns where a variable number of excitable orbits coexist at arbitrary positions within a time delay is possible only for a limited range of the bias parameter *β*. We start by considering the extend of this multi-stable region and denote its boundaries as *β* ∈ [*β*^*h*^, *β*^*SN*^]. In presence of delayed feedback, we notice in Fig. 3 that for *β* < *β*^*h*^ = 1.018 only the fully developed temporal pattern with 7 equi-spaced bits remains as the stable solution. We identified that this lowest limit *β*^*h*^ corresponds to the Andronov-Hopf bifurcation towards self-pulsation that already appears without feedback. This bifurcation corresponds to the point where the steady state solution becomes unstable and where one enters the oscillating regime that is the precursor of the canard explosion. The presence of delayed feedback slightly shifts this bifurcation point from the unperturbed value *β* = β^*^ so that we have that *β*^*h*^ = β^*^ only when *η* = 0. In the regime where *β* < *β*^*h*^ the only possible states are the ones with the largest number of elements within the round-trip since the instability of the background impedes the existence of empty uniform regions. Hence, for *β* < *β*^*h*^, the FHN with time delay behaves as a “normal” system operating on a monostable limit cycle.

On the other hand, the maximal value of *β* above which only the uniform state persists is governed by another mechanism. Here, we notice that with *η* = 0.18, only the uniform state subsists for values of the bias *β* > *β*^*SN*^ = 1.3. This upper limit of the multi-stable region is governed by a clear physical phenomenon: the excitability threshold increases with the distance from the Andronov-Hopf located at *β*^*h*^. As such, the excitable threshold becomes eventually too large to be overcome by the perturbation induced by the delayed re-injection of the excitable orbit, scaled by a factor *η*. Mathematically, this corresponds to the appearance and disappearance of these periodic orbits as Saddle Node Bifurcations of limit cycles.

The full bifurcation diagram of all the multi-LS solutions was obtained with DDE-BIFTOOL^54^ and is depicted in (Fig. 7b). To the best of our knowledge, this represents the first reconstruction of the bifurcation diagram of temporal Localized Structures in a delayed system.

We notice first that the simple bifurcation scenario found without feedback changes dramatically with *η* ≠ 0. The dominant periodic branch that corresponds to the canard blow-up (in black) develops a large number of folds as apparent in (Fig. 7b). We stress that only the upper part of this folded branch is stable and that at, e.g. at *β* = 1.1, it corresponds to the solution with a maximal number of LS within the time delay, i.e. the trace depicted in (Fig. 3d) with *N* = 7. By analogy with the terminology of spatially extended systems we denote this particular temporal trace without empty regions as the fully developed pattern. In this regime all the *N* = 7 pulses are interacting via their tails and can hardly be considered as independent. For instance, erasing an individual pulse would result in a smooth rearrangement of the other *N* = 6 remaining LS in order to minimize their residual repulsive interactions.

*A priori*, the continuation of the other solutions with a lower number of element would not have been impossible for several reasons. First, the periodic solutions where several LS coexist and are interleaved by long “empty” uniform regions like in (Fig. 3c) result in a very badly conditioned system for the bifurcation tracking. This is due to the presence of several quasi-neutral modes that can be traced back to the independent translation of each LS. In addition, these other branches of solutions do not stem as Andronov-Hopf bifurcations from a primary steady state branch as it is the case for the fully developed temporal pattern found here with *N* = 7. The other branches usually form isolae and occur as saddle-node bifurcation of limit cycles that are hardly detectable with standard bifurcation techniques.

The former difficulty stemming from the presence of several quasi-neutral modes was treated in the following manner. Among the infinity of different solutions with *N* LS (i.e. differing only in the *N* − 1 relative positions of the *N* LS) and period *T* ~ *τ*, we followed the *particular* branch where the *N* LS are equi-spaced. As a consequence, this particular solution is isomorphic to a solution with a single LS, yet with a period *T* ~ *τ*/*N*. This method impedes the arbitrary relative motion between the LS within the round-trip and we found that it resulted in a much easier continuation of the solution branches.

We circumvented the later difficulty by jump-starting DDEBIFTool with approximations of the temporal profiles obtained from direct temporal integration. Although cumbersome due to the weak convergence of the Newton method in a very high dimensional space, such a method allowed us to reconstruct, a full bifurcation diagram of all the multiple LS solutions that are depicted in colors in (Fig. 7b).

For an extensive study as a function of (*η*,*τ*,*β*), we have been able to identify two different scenarios that we summarize here. Some branches of solution like e.g. the one with *N* = 6 in (Fig. 7b) in red, can be disconnected from the rest of the web of solution. As previously mentioned, they form isolae and appear as hardly detectable saddle-node bifurcation of limit cycles.

**Figure 8:**
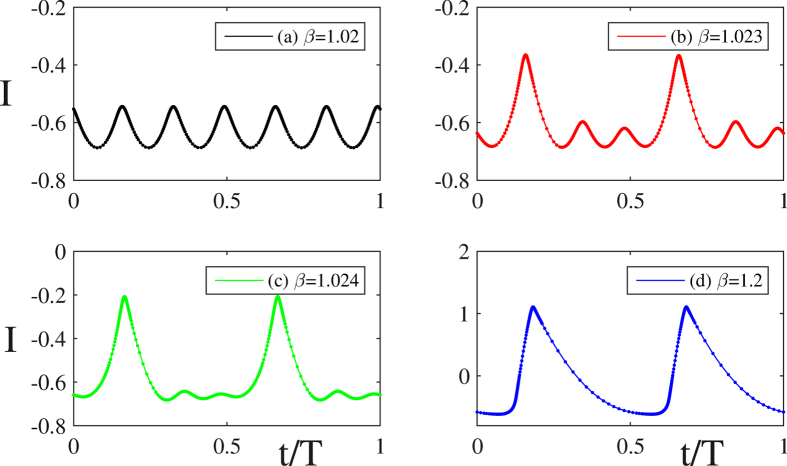
Simulated temporal profiles for the slow variable *I* as a function of the bias parameter *β*. The panels (**b**–**d**) correspond to the gradual transformation of the temporal profile as a function of the distance from the resonant Neimark-Sacker bifurcation over the primary branch depicted in (**a**). For clarity, the value of the time delay was set to *τ* = 200. The other parameters are *η* = 0.18.

Some other branches, like e.g. the one with *N* = 4 occurs as resonant Neimark-Sacker bifurcations over an unstable portion of the principal branch (here in black and leading to the fully developed pattern with *N* = 7 LS). We depict in (Fig. 8) the details of this behavior. At the bifurcation point, the unstable primary branch possesses in this case only 6 oscillations within the period, the latter being always close to the value of the time delay *τ*, see Fig. 8a. This periodic solution becomes unstable with respect to a temporal modulation whose period is one half of the repetition *τ*. This effect results in a periodic train favoring one element every three, see (Fig. 8b). Upon continuation of such a a quasi-periodic branch, two every three peaks gradually disappear, see (Fig. 8c), leading eventually to a solution with only *N* = 2, see (Fig. 8d). This mechanism for the generation of multiple LS presents a strong analogy with the secondary bifurcations occurring in spatially extended systems.

**Figure 9:**
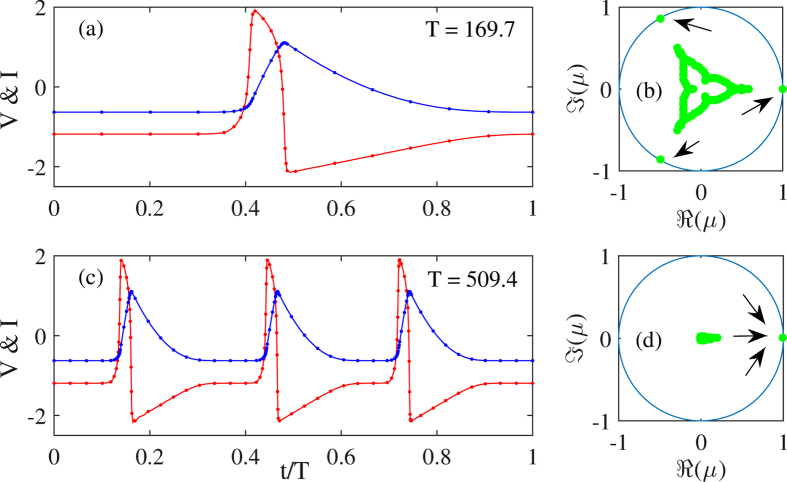
Simulated temporal profiles of two *τ*-periodic solutions with *N* = 3 equi-spaced LS using two different representations. In (**a**) we assume a temporal profile with a single LS and a period *T* ~ *τ*/3 while in (**b**) we consider three LS. The stability analysis of these two coexisting solutions is depicted in the panels (**b**,**d**) While the solution with a single LS possesses three Floquet multipliers with a threefold symmetry, the solution with *N* = 3 LS exhibit three multipliers close to unity.

Finally, we performed the stability analysis of the periodic solutions with a variable number of LS. As expected, we found that the solutions with *N* LS exhibited *N* quasi-neutral Floquet multipliers close to *μ* = 1, see (Fig. 9a,b). The inspection of the associated eigenvector showed that these *N* neutral modes correspond to the independent translation of each and every LS present within a time delay, demonstrating the independence of the various LS as in^30^. Interestingly, we note that the “reduced” periodic solutions that consist in a single LS but with a period *τ*/*N* does not show these degenerated Floquet multipliers close to unity, see (Fig. 9b). This is in agreement with the fact that by considering such a reduced temporal profile with a single element we removed the degenerated relative translation modes. Yet a strong signature of the fact that these solutions are not “simple” limit cycles remains since *even* the solution with a single LS and a period *τ*/*N* contains *N* complex Floquet Multipliers of unit modulus but whose phases are distributed evenly over the unit circle as *μ*_*j*_ = exp(2*πij*/*N*) with *j* ∈ [1, ···, *N*], see (Fig. 9d). This fact demonstrates that the reduced solution remains marginally stable against *N* different kind of perturbations.

## Discussion

In conclusion, we investigated a biologically inspired photonic memory based upon a delayed feedback regenerative oscillator. We reduce our proof-of-concept experimental system to the paradigmatic FitzHugh-Nagumo model complemented with a self-feedback autaptic connection, which bridges our physical photonic system with the biological neuron. We disclose the existence of a unique temporal response characteristic of localized structures in our photonic memory enabling an ideal support for bits in a random access memory for storage and reshaping of data information at high-bit-rate optical signals. Since the biological inspired photonic memory employs light at telecommunications wavelengths, we anticipate the development of fast neuron-inspired optical storage interconnects and signal processing applications. Although the present proof-of-concept photonic memory operates in the Mb/s range, lethargic times at tens of ps can be achieved by reducing the parasitics of the hybrid integrated circuit^39^,^45^. This will dramatically increase the attainable bit rate up to Gb/s as predicted by the simulations, paving the way for a new class of high-speed neural-inspired photonic memories.

## Methods

### Setup details

The experimental realization of the regenerative memory consists of an optoelectronic feedback loop containing a c.w. laser diode, an optical fiber delay line, and an RTD-PD. An erbium doped fiber amplifier (EDFA) is employed in the loop to compensate the losses of coupling and decoupling light from the RTD-PD and LD chips but can be avoided employing RTD-PD layer structures optimized for improved photo-detection response (estimated to be <0.2 A/W). The RTD-PD layer structure currently used consists of a 10 nm wide undoped AlAs/InGaAs/AlAs double barrier quantum well (DBQW) structure. The epitaxial layers include two InGaAlAs regions surrounding the DBQW that act as light absorbing layers for wavelengths around 1.55*μ*m. The LD device was a InGaAsP multi-quantum-well active region with an InP:Fe doped uncoated buried heterostructure and ridge mesas, with centre wavelength emission at ~1.55 *μ*m, threshold current ~6 mA^39^, and 3-dB modulation bandwidth in excess of 10 GHz.

In this work, for purposes of demonstration and experimental convenience, the data signals were injected electrically using a bias-T. The binary-coded data streams were generated using an arbitrary function generator (Tektronix AFG3251C). The signals could be also injected optically, taking advantage of the optical input port of the RTD-PD, and therefore buffering can be achieved using either electrical or optical incoming data. By operating the system in the excitable regime close to the NDR region, one would be able to store and regenerate optical bits of information in the fibre, the empty region signaling the “0” bits and the excitable optoelectronic pulses the “1”. When these bits are re-injected into the RTD-PD they trigger the generation of a new excitable cycle. This regenerative mechanism occurs after each round trip in the fibre which is extremely robust since even if the bit sequence is strongly deteriorated the all-or-none response of the excitable RTD-PD allows for a perfect regeneration. We employed a very long optical fiber loop providing 46 *μ*s cavity round-trip time, *τ*. The time *τ* was chosen in order that the memory buffer is much larger than the typical excitable lethargic time, *T*_*l*_, of the RTD-PD-LD excitable system, where in this case the measured *T*_*l*_ is around 500 ns.

### Theoretical model

We have analyzed our experimental results in the framework of delayed feedback nonlinear dynamical model systems employing a Liénard equation describing the RTD-PD excitable oscillator^44^,^45^ coupled to the single mode rate equations modeling the laser intensity and its population inversion. Regeneration is induced in such dynamical system by a delayed feedback term into the current source of the RTD-PD proportional to the intensity of the laser output. The Liénard oscillator-laser diode dynamical system is given by the following dimensionless coupled delay differential equations (DDEs):


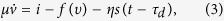



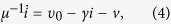



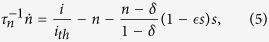



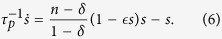


Equations (3)–(6), 8, 7, 8 represent the system of equations of the RTD-PD-LD with optical feedback control through the variable *s*(*t* − *τ*_*d*_) where *τ*_*d*_ is the time-delay with respect to the dimensionless time *t*; time is normalized to the characteristic *LC* resonant tank frequency, 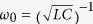, hence *τ* = *ω*_0_*t*. The feedback strength *η* parameter depends on RTD-PD detection characteristics and the fraction of the laser optical output power re-injected into the delayed feedback loop. Equations (3)–(4), 8 represent the Liénard oscillator where *v* and *i* are the dimensionless voltage and current variables, respectively. The function *f*(*v*) describes the nonlinear I-V curve, *R* = *γ*(*I*_0_/*V*_0_), and 
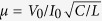
 is a dimensionless parameter.

Equations (5)–(6), 8 are the dimensionless rate equations describing LD normalized photon *s*(*t*) and injected carrier *n*(*t*) densities. The charge carrier *n*(*t*) in Eq. (5) is normalized to threshold providing that *δ* = *N*_0_/*N*_*th*_, where *N*_0_ is the carrier density for transparency, and *N*_*th*_ is the threshold carrier density; 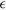 stands for the dimensionless laser gain saturation. The dimensionless laser diode threshold current is *i*_*th*_; The parameters *τ*_*n*_ and *τ*_*p*_ come from the time rescaling.

The predictions of the model are in very good agreement with the experimental results allowing to estimate a theoretical limit of operation of several Gb/s for such buffer configuration. In the limit case where the pulses are broad and the dynamics is slow as compared to the relaxation oscillation frequency of the laser, it is possible to adiabatically eliminate the equations for (*s*, *n*). As such the delayed intensity *s*(*t* − *τ*) becomes proportional to the current of the RTD-PD device *i*(*t*). As a last step, the nonlinear function *f*(*v*) has to be expanded in Taylor series around the center of the NDR. Neglecting the second order term yielding the asymmetry of *f*(*υ*) in the expansion and cutting the expansion to third yields the FHN equations presented in the main manuscript, after further trivial rescaling.

### Numerical Methods

Eqs (1) and (2) were numerically integrated with a fourth-order Runge-Kutta method with constant step size (*h* = 10^−2^)^55^. The delayed contributions in Eqs (1) and (2) demand a special care. To advance the solution with a step *h* from *t*_*n*_ = *nh* to *t*_*n*+1_, the Runge-Kutta algorithm requires the values of *I*(*t* − *τ*) at intermediate points *t*_*n*+1/2_. These are not known and must be interpolated from past values with an order of approximation consistent with that of the algorithm of integration. Therefore, besides keeping memory of the past values of *I*(*t*) we also retain the past values of the time derivative 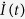. Such a method allows building a third order Hermite polynomial approximation for *I*(*t*) between the time (*t*_*n*_ − *τ*) and (*t*_*n*+1_ − *τ*). By evaluating this interpolating polynomial at (*t*_*n*+1/2_ − *τ*), we ensure an overall fourth order accuracy. Finally, the stochastic noise contribution in Eqs (1, 2) is added after the deterministic step by simply using the Euler method^55^ and scaling *ξ* by 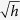.

Due to the infinite dimensionality of DDEs, an initial condition must be given over an interval *t* ∈ [−*τ*, 0]. Localized Structures can be generated numerically by starting with a uniform initial condition corresponding to the steady sate solution (*V*_*s*_, *I*_*s*_) over an interval *t* ∈ [−*τ*, 0] in which we insert, for the sake of simplicity, a rectangular perturbation. This perturbation within the memory buffer that corresponds to the delayed values of *I*(*t* − *τ*) acts as a seed for the regenerative periodic orbit. In agreement with the basic properties of excitable systems, as long as its duration is shorter than the lethargic time *T*_*l*_, the precise form of the perturbation is mostly irrelevant and a regenerative excitable orbit is generated if the integral, i.e. the “energy”, of the rectangular perturbation exceeds the excitable threshold. In this case, this initial perturbation results in a single LS in the asymptotic regime getting periodically regenerated at each round-trip. From this newly found periodic solution, additional perturbations can be applied in order to generate additional independent LS if they are properly timed, although Several LS can also be generated in a single event using multiple rectangular perturbations.

## Additional Information

**How to cite this article**: Romeira, B. *et al.* Regenerative memory in time-delayed neuromorphic photonic resonators. *Sci. Rep.* **6**, 19510; doi: 10.1038/srep19510 (2016).

## References

[b1] Loos H. V. D. & Glaser E. M. Autapses in neocortex cerebri: synapses between a pyramidal cell’s axon and its own dendrites. Brain Research 48, 355–360 (1972). 4645210 10.1016/0006-8993(72)90189-8

[b2] Herrmann C. S. & Klaus A. Autapse turns neuron into oscillator. International Journal of Bifurcation and Chaos 14, 623–633 (2004).

[b3] Flight M. H. Neuromodulation: Exerting self-control for persistence. Nat Rev Neurosci 10, 316–316 (2009). 10.1038/nrn2637 19382325

[b4] Branco T. & Staras K. The probability of neurotransmitter release: variability and feedback control at single synapses. Nat Rev Neurosci 10, 373–383 (2009). 19377502 10.1038/nrn2634

[b5] Jo S. H. *et al.* Nanoscale Memristor Device as Synapse in Neuromorphic Systems. Nano Letters 10, 1297–1301 (2010). 20192230 10.1021/nl904092h

[b6] Indiveri G. *et al.* Neuromorphic silicon neuron circuits. Frontiers in neuroscience 5 (2011). 10.3389/fnins.2011.00073 PMC3130465 21747754

[b7] Merolla P. A. *et al.* A million spiking-neuron integrated circuit with a scalable communication network and interface. Science 345, 668–673 (2014). 25104385 10.1126/science.1254642

[b8] Tait A., Nahmias M., Shastri B. & Prucnal P. Broadcast and weight: An integrated network for scalable photonic spike processing. Lightwave Technology, Journal of 32, 4029–4041 (2014).

[b9] Kuzum D., Jeyasingh R. G. D., Lee B. & Wong H. S. P. Nanoelectronic programmable synapses based on phase change materials for brain-inspired computing. Nano Letters 12, 2179–2186 (2011). 21668029 10.1021/nl201040y

[b10] Burić N. & Todorović D. Dynamics of Fitzhugh-Nagumo excitable systems with delayed coupling. Phys. Rev. E 67, 066222 (2003). 10.1103/PhysRevE.67.066222 16241341

[b11] Stepan G. Delay effects in brain dynamics. Philosophical Transactions of the Royal Society A: Mathematical, Physical and Engineering Sciences 367, 1059–1062 (2009). 10.1098/rsta.2008.0279 19218150

[b12] Yacomotti A. M. *et al.* Coupled optical excitable cells. Phys. Rev. E 66, 036227 (2002). 10.1103/PhysRevE.66.036227 12366244

[b13] Schöll E., Hiller G., Hövel P. & Dahlem M. A. Time-delayed feedback in neurosystems. Philosophical Transactions of the Royal Society of London A: Mathematical, Physical and Engineering Sciences 367, 1079–1096 (2009). 10.1098/rsta.2008.0258 19218152

[b14] Kelleher B., Bonatto C., Skoda P., Hegarty S. P. & Huyet G. Excitation regeneration in delay-coupled oscillators. Physical Review E 81, 036204 (2010). 10.1103/PhysRevE.81.036204 20365829

[b15] Weicker L., Erneux T., Keuninckx L. & Danckaert J. Analytical and experimental study of two delay-coupled excitable units. Phys. Rev. E 89, 012908 (2014). 10.1103/PhysRevE.89.012908 24580298

[b16] Ikeda K. Multiple-valued stationary state and its instability of the transmitted light by a ring cavity system. Optics Communications 30, 257–261 (1979).

[b17] Neyer A. & Voges E. Dynamics of electrooptic bistable devices with delayed feedback. Quantum Electronics, IEEE Journal of 18, 2009–2015 (1982).

[b18] Aida T. & Davis P. Oscillation modes of laser diode pumped hybrid bistable system with large delay and application to dynamical memory. Quantum Electronics, IEEE Journal of 28, 686–699 (1992).

[b19] Giacomelli G. & Politi A. Relationship between delayed and spatially extended dynamical systems. Phys. Rev. Lett. 76, 2686–2689 (1996). 10060763 10.1103/PhysRevLett.76.2686

[b20] Coullet P., Riera C. & Tresser C. A new approach to data storage using localized structures. Chaos 14, 193–201 (2004). 15003061 10.1063/1.1642311

[b21] Umbanhowar P. B., Melo F. & Swinney H. L. Localized excitations in a vertically vibrated granular layer. Nature 793–796 (1996).8657283

[b22] Astrov Y. A. & Purwins H. Plasma spots in a gas discharge system: birth, scattering and formation of molecules. Physics Letters A 283, 349–354 (2001).

[b23] Niedernostheide F. J., Arps M., Dohmen R., Willebrand H. & Purwins H. G. Spatial and spatio-temporal patterns in pnpn semiconductor devices. Physica Status Solidi (B) 172, 249–266 (1992).

[b24] Lee K.-J., McCormick W. D., Pearson J. & Swinney H. L. Experimental observation of self-replicating spots in a reaction-diffusion system. Nature 369, 215–218 (1994).

[b25] Wu J., Keolian R. & Rudnick I. Observation of a nonpropagating hydrodynamic soliton. Phys. Rev. Lett. 52, 1421–1424 (1984).

[b26] Moses E., Fineberg J. & Steinberg V. Multistability and confined traveling-wave patterns in a convecting binary mixture. Phys. Rev. A 35, 2757–2760 (1987). 9898473 10.1103/physreva.35.2757

[b27] Barland S. *et al.* Cavity solitons as pixels in semiconductor microcavities. Nature 419, 699–702 (2002). 12384692 10.1038/nature01049

[b28] Hachair X. *et al.* Cavity solitons in a driven VCSEL above threshold. Selected Topics in Quantum Electronics, IEEE Journal of 12, 339–351 (2006).

[b29] Leo F. *et al.* Temporal cavity solitons in one-dimensional Kerr media as bits in an all-optical buffer. Nat Photon 4, 471–476 (2010).

[b30] Garbin B., Javaloyes J., Tissoni G. & Barland S. Topological solitons as addressable phase bits in a driven laser. Nat. Com. 6 (2015). 10.1038/ncomms6915 25557181

[b31] Nicolis G. & Prigogine I. Self-Organization in Nonequilibrium Systems: From Dissipative Structures to Order through Fluctuations (Wiley, 1977).

[b32] Descalzi O., Clerc M., Residori S. & Assanto G. Localized States in Physics: Solitons and Patterns, vol. 751 of Lecture Notes in Physics (Springer Berlin Heidelberg, 2011).

[b33] Marino F., Giacomelli G. & Barland S. Front pinning and localized states analogues in long-delayed bistable systems. Phys. Rev. Lett. 112, 103901 (2014). 24679295 10.1103/PhysRevLett.112.103901

[b34] Samardak A. *et al.* Spiking computation and stochastic amplification in a neuron-like semiconductor microstructure. Journal of Applied Physics 109 (2011).

[b35] Barbay S., Kuszelewicz R. & Yacomotti A. M. Excitability in a semiconductor laser with saturable absorber. Optics letters 36, 4476–4478 (2011). 22139214 10.1364/OL.36.004476

[b36] Selmi F. *et al.* Relative refractory period in an excitable semiconductor laser. Phys. Rev. Lett. 112, 183902 (2014). 24856697 10.1103/PhysRevLett.112.183902

[b37] Peil M., Jacquot M., Chembo Y. K., Larger L. & Erneux T. Routes to chaos and multiple time scale dynamics in broadband bandpass nonlinear delay electro-optic oscillators. Phys. Rev. E 79, 026208 (2009). 10.1103/PhysRevE.79.026208 19391821

[b38] Romeira B., Kong F., Figueiredo J., Javaloyes J. & Yao J. High-speed spiking and bursting oscillations in a long-delayed broadband optoelectronic oscillator. Lightwave Technology, Journal of 33, 503–510 (2015).

[b39] Romeira B. *et al.* Excitability and optical pulse generation in semiconductor lasers driven by resonant tunneling diode photo-detectors. Opt. Express 21, 20931–20940 (2013). 24103966 10.1364/OE.21.020931

[b40] Hodgkin A. L. & Huxley A. F. A quantitative description of membrane current and its application to conduction and excitation in nerve. Journal of Physiology 117, 500–544 (1952). 12991237 10.1113/jphysiol.1952.sp004764 PMC1392413

[b41] FitzHugh R. Mathematical models of threshold phenomena in the nerve membrane. The bulletin of mathematical biophysics 17, 257–278 (1955).

[b42] Nagumo J., Arimoto S. & Yoshizawa S. An active pulse transmission line simulating nerve axon. Proceedings of the IRE 50, 2061–2070 (1962).

[b43] Liénard A.-M. Etude des oscillations entretenues. Revue générale de l'électricité 23, 901–912 and 946–954 (1928).

[b44] Romeira B. *et al.* Delayed feedback dynamics of Liénard-type resonant tunneling-photo-detector optoelectronic oscillators. Quantum Electronics, IEEE Journal of 49, 31–42 (2013).

[b45] Romeira B. *et al.* Stochastic induced dynamics in neuromorphic optoelectronic oscillators. Optical and Quantum Electronics 1–6 (2014).

[b46] Hodgkin A. L., Huxley A. F. & Katz B. Measurement of current-voltage relations in the membrane of the giant axon of loligo. The Journal of physiology 116, 424 (1952). 14946712 10.1113/jphysiol.1952.sp004716 PMC1392219

[b47] Pyragas K. Continuous control of chaos by self-controlling feedback. Physics Letters A 170, 421–428 (1992).

[b48] Tyson J. J. & Keener J. P. Singular perturbation theory of traveling waves in excitable media (a review). Physica D: Nonlinear Phenomena 32, 327–361 (1988).

[b49] Izhikevich E. M. Dynamical systems in neuroscience : the geometry of excitability and bursting. Computational neuroscience (MIT Press, Cambridge, Mass., London, 2007).

[b50] Marconi M., Javaloyes J., Balle S. & Giudici M. How lasing localized structures evolve out of passive mode locking. Phys. Rev. Lett. 112, 223901 (2014). 24949767 10.1103/PhysRevLett.112.223901

[b51] Benoît E., Callot J. L., Diener F. & Diener M. Chasse au canard (première partie). Collectanea Mathematica 32 37–74 (1981).

[b52] Keener J. & Sneyd J. Mathematical Physiology: I: Cellular Physiology, vol. 1 (Springer, 2008).

[b53] Meron E. Pattern formation in excitable media. Physics Reports 218, 1–66 (1992).

[b54] Engelborghs K., Luzyanina T. & Samaey G. Dde-biftool v. 2.00: *a Matlab package for bifurcation analysis of delay differential equations*. Tech. Rep., Department of Computer Science, K.U.Leuven, Belgium. (2001). http://twr.cs.kuleuven.be/research/software/delay/ddebiftool.shtml

[b55] Press W. H., Teukolsky S. A., Vetterling W. T. & Flannery B. P. Numerical Recipes: The Art of Scientific Computing (Cambridge University Press, 2007).

